# Where Do People in Nigeria Get Their Contraception?

**DOI:** 10.1371/journal.pmed.0020366

**Published:** 2005-11-29

**Authors:** Oladapo A Ladipo

## Abstract

Knowing where users obtain different contraceptive methods is useful for planning service delivery, argues Ladipo, who examines a new study on contraception in Nigeria.

In this issue of *PLoS Medicine*, Oye-Adeniran and colleagues report a new community study in Nigeria in which contraceptive users were surveyed about their sources of family planning information and contraception [1]. Enquiry about the source of contraceptive is a standard part of family planning surveys that assess knowledge, attitudes, and practices, and these surveys are commonly used in Nigeria and elsewhere [2,3]. The underlying proposition is that knowing where users obtain different contraceptive methods is useful for planning service delivery. The associated question—why do people choose a particular source for obtaining contraceptives (for example, a pharmacy rather than a clinic)—generally receives only passing attention.

Over the years, there has been little variation in the pattern of contraceptive sourcing. Nonclinic facilities are the main source for obtaining condoms and the oral contraceptive pill, while the intrauterine contraceptive device and injectable contraceptives—both of which require health provider intervention—are predominately obtained from the clinic setting. Over time, neither the clinic nor the nonclinic sources have developed to their full potential as family planning providers, despite years of family planning programming in both public and private sectors.

## The New Study

Oye-Adeniran and colleagues surveyed 2,001 persons aged 14–49 from four states of Nigeria—Anambra from the southeast, Oyo from the southwest, Kaduna from the northwest, and Bauchi from the northeast. These states were randomly selected for the study, one each from Nigeria's four health zones. A multistage cluster sampling design was used to select the respondents. Of the 2,001 people surveyed, 1,647 (82.3%) were sexually active, out of whom 244 were found to be using contraceptive methods at the time of the study, giving a contraceptive prevalence rate of 14.8%.

Knowing where users obtain different contraceptive methods is useful for planning service delivery.

The study had three major findings: (1) friends are the predominant source of information on contraception; (2) young people tend to prefer chemists (pharmacists), while older people prefer government and private hospitals as sources of contraception; and (3) Catholics prefer to avoid public health institutions. These findings are similar to those obtained by Nigeria's 2003 Demographic and Health Survey [4].

## What Issues Does the Study Raise?

There are several issues arising from this study. First, sources of contraception in the public sector are not set up to work with nonclinic sources, although a referral system may well suit the needs of clients. Second, the roles of the limited number of modern contraceptive methods available and of service providers in a sexually active person's choice of contraceptive method are not yet fully resolved [5,6]. Third, the cultural disposition against providing contraceptives to young people in Nigeria, in spite of their sexual activity, makes the young wary of public health institutions. At such institutions, they are likely to confront adult health providers who will often frown on their sexual activity. The added advantage of the chemist for both young people and Catholics may well be connected to their need for anonymity, which the chemist provides, but which public health institutions, with their formal procedures for documentation, counseling, and service provision, deny. Above all, the findings reinforce the point that clinics at all levels of the health delivery system are characterized by unfriendliness to youths.

A major limitation of Oye-Adeniran and colleagues' study is the relatively small number of respondents who were using contraceptive methods. This limits extrapolation of the study to the general population, which may have different demographic and socioeconomic characteristics than the study sample.

## How Can We Reach Young People?

It is obvious that the provision of youth-friendly clinics still eludes both policymakers and service providers. The cultural predisposition against family planning services being made available to youths is a major barrier to reaching young people. Until the sexual activity and associated risks among young people are acknowledged by the adult population, and the provision of contraceptives is seen as a solution rather than the problem, the implementation of youth-friendly clinics and their utilization will be compromised.

Although Oye-Adeniran and colleagues mentioned the preference of the adult married population for public sector services, there is as yet no clear identification of the level of satisfaction that this group derives from the services provided. The very presence of older clients at public clinics may well be a deterrent to youth patronage. The service characteristics of the chemists that make chemist shops such attractive locations for youths could reward in-depth study [7,8]. A likely direction of such investigation will be the extent to which the anonymity and lack of curiosity or the absence of intrusive counseling in chemist shops are preferred by young people to the formal procedures in public health institutions.
